# The Link between Thyroid Function and Depression

**DOI:** 10.1155/2012/590648

**Published:** 2011-12-14

**Authors:** Mirella P. Hage, Sami T. Azar

**Affiliations:** Division of Endocrinology and Metabolism, Department of Internal Medicine, American University of Beirut Medical Center, P.O. Box 11-0236, Riad El Solh, Beirut 1107 2020, Lebanon

## Abstract

The relation between thyroid function and depression has long been recognized. Patients with thyroid disorders are more prone to develop depressive symptoms and conversely depression may be accompanied by various subtle thyroid abnormalities. Traditionally, the most commonly documented abnormalities are elevated T4 levels, low T3, elevated rT3, a blunted TSH response to TRH, positive antithyroid antibodies, and elevated CSF TRH concentrations. In addition, thyroid hormone supplements appear to accelerate and enhance the clinical response to antidepressant drugs. However, the mechanisms underlying the interaction between thyroid function and depression remain to be further clarified. Recently, advances in biochemical, genetic, and neuroimaging fields have provided new insights into the thyroid-depression relationship.

## 1. Introduction

The association between thyroid function and psychiatric disorders particularly mood disorders has long been recognized. Historically, this association has been described more than 200 years ago. Parry in 1825 reported an increased incidence of “nervous affectations” in thyroid disorders. Gull in 1873 showed the relation between myxedema and psychosis that was confirmed in 1888 by the Committee of the Clinical Society. Later, Asher in 1949 coined the term “myxedema madness” to describe the mental state of subjects with hypothyroidism [1].

Today, it is well recognized that disturbances in thyroid function may significantly affect mental status including emotion and cognition. Both excess and insufficient thyroid hormones can cause mood abnormalities including depression that is generally reversible with adequate thyroid treatment. On the other hand, depression can be accompanied by subtle thyroid dysfunction. Overt thyroid disease is rare in depression. One to 4% of patients with affective disorders are found to have overt hypothyroidism while subclinical hypothyroidism occurs in 4% to 40% of these patients [2]. Furthermore, thyroid hormones are reported by many authors to be an effective adjunct treatment for depression.

In this paper, we will present an overview of thyroid hormone metabolism in the brain, reexamine the different observations and clinical studies assessing the relationship between thyroid and depression, and shed light on the advances in neuroimaging approaches in this field. Understanding the link between both disorders will guide clinicians to appropriately interpret thyroid function tests in depression, better understand the pathophysiology of both diseases, and try to identify the subjects who will benefit the most from thyroid supplementation.

## 2. Literature Search

A PubMed search was performed through the English literature from 1969 to present using the keywords: “acceleration,” “antidepressant treatments,” “augmentation,” “brain,” “depression,” “hyperthyroidism,” “hypothyroidism,” and “mood disorder.”

## 3. Overview of Thyroid Hormone Metabolism in the Brain

The hypothalamic-pituitary-thyroid axis (HPT) is a complex interplay between several factors: thyroid hormones, deiodinase enzymes, transporter proteins, and receptors. An understanding of the interactions of these factors may contribute to better elucidate the pathophysiology of psychiatric disorders as well as the response to psychiatric treatment.

The secretion of thyroid hormones is regulated by pituitary thyrotropin (TSH) which itself is stimulated by hypothalamic thyrotropin-releasing hormone (TRH) and downregulated by serum thyroid hormones. Twenty percent of triiodothyronine (T3) in the cerebral cortex is secreted directly by the thyroid while 80% is derived from local conversion of thyroxine (T4) by deiodination [3, 4]. Most of the T4 enter the brain via a number of transporters including transthyretin (TTR), a thyroid hormone transport protein synthesized by the choroid plexus and secreted into the cerebrospinal fluid [5, 6]. Deiodination occurs intracellularly mainly in glial cells and T4 must enter these cells through specialized plasma membrane carrier proteins including organic anion transporter polypeptide 1 (OATP1C1) and monocarboxylase transporter 8 (MCT8). The former preferentially transports T4 and rT3 while the latter is more specific for T3 transport [7]. In the glial cells, T4 is converted to T3 by the deiodinase enzyme type 2 (D2) while it is inactivated to 3,3′,5′-triiodothyronine (rT3) in the neuronal cells by the deiodinase enzyme type 3 (D3). The latter also deiodinates T3 into inactive T2. The actions of T3 are mediated by binding to thyroid hormone nuclear receptors (THRs). In the adult brain, THR-*α* is most highly expressed and constitutes 70–80% of THR distribution [8].

Thus, the HPT axis includes complex pathways and impairment in its components has been linked in some studies to behavioral changes as will be further pointed out.

## 4. Neuropsychiatric Manifestations of Thyroid Disorders

Primary thyroid disorders including both hypothyroidism and hyperthyroidism may be accompanied by various neuropsychiatric manifestations ranging from mild depression and anxiety to overt psychosis.

Dysphoria, anxiety, irritability, emotional lability, and impairment in concentration constitute the classical neuropsychiatric symptoms occurring in hyperthyroidism or thyrotoxicosis. However, elderly patients may present in a state mimicking a depressive disorder with apathy, lethargy, and pseudodementia [9]. Anxiety disorders have been found to occur in approximately 60% of hyperthyroid patients while depressive disorders occurred in 31 to 69% [10, 11].

On the other hand, hypothyroid patients frequently demonstrate features of depression, cognitive dysfunction, apathy, and psychomotor slowing. In severe forms of hypothyroidism, clinical symptoms may mimic that of melancholic depression and dementia [12]. However, there is less evidence on the association of subclinical hypothyroidism with cognitive dysfunction and affective disorders particularly depression although recently, a prevalence of 63.5% of depressive symptoms was reported in an Italian population with subclinical hypothyroidism. Nevertheless, therapy with levothyroxine alone was not sufficient to induce a total remission of depressive symptoms in this population [13]. Furthermore, Bauer et al. showed that TSH levels in hypothyroidism correlated with disease severity [14] and Joffe and Levitt found a disparity in depressive symptoms manifestations and severity among patients with a low-normal TSH versus those with a high-normal TSH. However, in the latter study no difference in treatment outcome was observed between the two groups [15]. In patients treated with T4, psychological symptoms may persist even when they achieve a euthyroid state [16]. Impaired psychological wellbeing in these subjects may be related to the occurrence of genetic polymorphisms in the D2 gene [17] as well as the OATPC1 encoding gene [18].

According to the American Association of Clinical Endocrinologists, “The diagnosis of subclinical or clinical hypothyroidism must be considered in every patient with depression” [19]. Indeed, among the various neuropsychiatric manifestations of thyroid disorders, depression remains the most common [20].

## 5. Thyroid Status in Patients with Depression

Several thyroid abnormalities have been associated with mood disorders particularly depression. However, the vast majority of patients with depression do not have biochemical evidence of thyroid dysfunction [21, 22]. When thyroid abnormalities exist, they consist mainly of elevated T4 levels, low T3, elevated rT3, blunted TSH response to TRH, positive antithyroid antibodies, and elevated cerebrospinal fluid (CSF) TRH concentrations. A state of brain hypothyroidism in the setting of systemic euthyroidism [23–25] has been suggested. This could result from a defect in the thyroid hormone receptor [26], or in the thyroid hormone transport and uptake into the brain and neuronal cells [7, 27]. Mice with receptor-mediated hypothyroidism caused by a heterozygous mutation in the THR*α*-1 have shown increased depressive behavior responsive to continuous T3 administration [26]. Furthermore, lower levels of CSF TTR have been reported in patients with depression compared to controls [28–30] resulting in a state of “brain hypothyroidism” with, nonetheless, normal peripheral thyroid hormones [28]. Mutations in MCT8 have also been recognized to cause isolated brain hypothyroidism by blocking T3 transport into neurons [8, 31].

## 6. Peripheral Thyroid Hormone Concentrations

### 6.1. Thyroxine (T4)

Studies examining total and free plasmatic T4 levels in patients with depression have shown inconsistent results. Serum T4 levels in the upper range of normal or slightly higher have been reported in depressed patients as compared to healthy or psychiatric controls. These levels have been found to regress after successful treatment of depression [32].

One mechanism explaining the increase in T4 seen in depression is the activation of hypothalamic TRH producing neurons and subsequent increase in thyroid function secondary to the rise in cortisol associated with depression [19, 24, 33]. In addition, it has been shown that elevated serum T4 levels fall after successful treatment of depression. A direct effect of antidepressants on the TRH neuron has been demonstrated resulting in an inhibition of TRH secretion [34]. This suggests that the decrease in T4 levels with initiation of antidepressants could be secondary to a direct effect on TRH neuron and thus to a reduced stimulation of the thyroid axis.

Recently, the Caerphilly Prospective Study examined the link between thyroid function and minor psychiatric morbidity among 2269 middle-aged men. A weak positive association between total T4 and chronic psychiatric morbidity was observed after 12.3 years of followup. However, this was consistent with chance after adjustments were done for social class, alcohol, and smoking behaviors. On the other hand, meta-analyses of seven studies showed TSH to be negatively correlated with depression and total T4 to be positively related to a depressed mood [35].

### 6.2. Triiodothyronine (T3) and 3,3′,5′-Triiodothyronine (rT3)

In patients with depression and no other illnesses, a “low T3 syndrome” has been described [36]. In one study [37], a normal daily production of T3 was reported among unmedicated and moderately depressed patients. The combination of increased T4 yet normal T3 production supports the hypothesis of a reduced deiodination of T4 into T3 as seen in the euthyroid sick syndrome [37]. An elevated rT3 was also reported in association with unipolar depression [38] as demonstrated by Linnoila et al., and Kirkegaard and Faber found as well high rT3 levels in endogenous depression that normalized after electroconvulsive therapy [39]. It has been hypothesized that depression leads to inhibition of the D2 enzyme responsible for conversion of T4 into T3 due to the increase in cortisol levels [40]. This favors the production of rT3 by D3 enzyme. In addition, elevated levels of rT3 were found in CSF of patients with unipolar depression [41].

## 7. Antithyroid Antibodies

A prevalence of up to 20% of elevated titers of antithyroid antibodies has been documented in depressed patients in several reports compared to a 5–10% prevalence in the general population [42–45]. However, this should be viewed with caution since these reports either lacked a control group [42, 43, 45] or showed no significant difference between the group with an affective disorder and the control group with a nonaffective psychiatric disorder [44]. Furthermore, whether this link may have any clinical significance remains unclear since it was most often accompanied by normal serum TSH concentrations [42, 43]. In addition, Fountoulakis et al. found higher thyroid binding inhibitory immunoglobulins (TBII) in depressed patients suggesting the presence of an autoimmune process involving the thyroid gland in depressed patients [46].

## 8. Blunted TSH Response and Abnormal Diurnal Rhythm

Depression has been linked to various endogenous circadian rhythms abnormalities such as diurnal mood variation, abnormalities in core body temperature, cortisol secretion, and sleep-wake cycle [47]. In addition to these circadian dysfunctions, depression has been linked to an abnormal diurnal TSH rhythm as well. An absent TSH nocturnal surge [48] has been noted in depression and a lower basal TSH has been reported in major depression as opposed to nonmajor depression [49]. Furthermore, a blunted TSH response to TRH was reported in about 25–30% of depressed subjects compared to healthy ones [50–54]. One preeminent hypothesis to explain the above finding is that chronic TRH hypersecretion associated with depression leads to downregulation of pituitary TRH receptors [24, 53, 55, 56]. In support to this hypothesis are reports of elevated CSF concentrations of TRH in depressed patients [57, 58].

The prolonged release of TRH in depression may be seen as a compensatory response to the decreased 5HT activity in an attempt to normalize 5HT function and maintain normal levels of thyroid hormones [59]. An alternative explanation is that the blunted TSH response may be induced by the hypercortisolism associated with depression or the elevated thyroid hormone levels mediated by adrenergic mechanisms [60, 61].

In addition, TRH has been postulated in early studies to have an antidepressant effect.

Administration of TRH at a dose of 500 *μ*g parenterally to unipolar depressed women led to a significant improvement in depression ratings [50, 51]. Furthermore, TRH-R1 knockout mice showed increased anxiety and depression-like behavior thus supporting a role for endogenous TRH in mood regulation [62]. The extent to which the endogenous TRH system is involved in mood regulation and the underlying implicated mechanisms remain to be defined.

## 9. Thyroid Hormone Supplementation in Depression

Thyroid hormones have been used as an adjunct to antidepressant therapy since the late 1960s to accelerate clinical response to antidepressants (acceleration) and to potentiate clinical response in non-responders to antidepressants (augmentation).

An acceleration of antidepressant effect by T3 has been initially shown more than 30 years ago in several reports [63–66]. A meta-analysis of these early double-blind placebo controlled trials concluded that T3 was effective in accelerating the clinical response to tricyclic antidepressants in patients with nonrefractory depression. The effects of T3 acceleration appeared to be more remarkable as the percentage of women in a trial increased therefore suggesting that women might benefit more than men from T3 supplementation [67]. In addition, several reports examined the role of T3 as an augmentation strategy to antidepressants in refractory depression. The majority involved the use of tricyclic antidepressants and supported the role for T3 in managing refractory depression [68]. More recently, studies assessing the newer and more tolerable antidepressant agents, the selective serotonin reuptake inhibitors (SSRIs) in combination with T3 have yielded confounding results. A meta-analysis of these studies concluded that simultaneous initiation of T3 and SSRI is not significantly more likely to accelerate or enhance the clinical response in depressed patients compared to SSRI monotherapy. However, the authors suggested that T3 and SSRI cotherapy may be effective in a subset of depressed patients including those with atypical depression, or those with functional D1 gene polymorphism [69]. As previously described, D1 has a key role in T4 to T3 conversion. In a study by Cooper-Kazaz et al. patients with C785T polymorphism in D1 gene had a better response to T3 augmentation therapy. Therefore, depressed patients with genetically determined lower T4 to T3 conversion could therefore derive more benefit from thyroid hormone augmentation therapy [70].

Fewer studies assessed the efficacy of T4 in the treatment of affective disorders. Joffe and Singer found a significantly higher response to tricyclic antidepressants with T3 (53%) compared to T4 (19%) [71]. However, use of T4 in supraphysiological doses to treatment-resistant unipolar and bipolar depression was effective in approximately 50% of patients as reported by Baumgartner in a review of eight open clinical trials (*N* = 78) [72]. Surprisingly, T4 in high doses was well tolerated even in patients treated for up to 51 months. However, in healthy subjects, supraphysiological T4 doses were less well tolerated due to higher increments in thyroid hormones after supplementation [73]. A possible explanation would be a greater inactivation of T4 to rT3 in depressed patients compared to healthy subjects [74].

Clearly, further research is needed to ascertain whether thyroid hormone supplementation may effectively accelerate and potentiate therapeutic response to antidepressant drugs. In addition, the role of genetic variations in deiodinase enzymes in the response to antidepressive therapy merits further investigation.

## 10. Effect of Depression Treatment on Thyroid Status

Normalization of pretreatment thyroid function tests mainly T4 levels with remission of depression has been reported [75, 76]. Whether this is related to clinical recovery or merely a result of a direct effect of antidepressants remains to be determined.

Both tricyclic antidepressants [77] and SSRIs [78] appear to enhance the activity of D2 resulting in an increased conversion of T4 into active T3 in the brain. T3 was suggested to enhance neurotransmission in the central noradrenergic pathways [79] and deficiency in catecholamines has been raised as a possible mechanism in depression [80]. Additionally, it has been shown that anti-depressants with variable mechanisms of action have different effects on thyroid indices [81]. Further studies are therefore required to better elucidate this complex interaction between the HPT axis and the neurotransmitter system.

## 11. Association of Depression with Postpartum Thyroid Disease

The association of postpartum depression with postpartum thyroiditis or with positive thyroid antibodies is still not well defined. Early studies noted a minor association between thyroid dysfunction and postnatal depression [82]. More recently, a higher frequency of mild to moderate depression was observed in postpartum female subjects with positive antithyroid antibodies regardless of thyroid function [83–85]. However, an attempt to decrease the incidence of postpartum depression in thyroid antibody positive women with daily administration of thyroxine for 18 weeks postpartum was unsuccessful [86].

## 12. Neuroimaging in Thyroid and Mood Disorders

A few studies have been performed to assess the changes in cerebral perfusion and metabolism in patients with hypothyroidism particularly those with Hashimoto's thyroiditis or status post-thyroidectomy for thyroid carcinoma.

Some have reported diffuse global hypoperfusion [87–89] while others demonstrated decreased regional cerebral blood flow [90–92]. Furthermore, variable findings regarding restoration of blood flow with treatment have been documented. While some demonstrated at least partial normalization of cerebral blood flow [14, 87, 89] others found persistent hypoperfusion with restoration of the euthyroid state [90–92].

The inconsistency in the above findings can be accounted for by the variability in the degree of hypothyroidism and the differences in the etiology and duration of the disease in the various studied populations.

Studies assessing cerebral blood flow and metabolism in depression are more numerous. The most widely replicated finding from these studies is hypoperfusion in anterior cortical structures [93, 94] that was reversible after psychotherapy and pharmacotherapy [95–97]. In addition to frontal hypoperfusion, increased perfusion has been observed in various limbic regions most notably the amygdala [93].

In a study comparing cerebral blood flow in hypothyroidism and major depression, hypothyroid patients exhibited hypoperfusion in posterior aspects of the brain in contrast to an anterior cerebral hypoperfusion in depressed patients. Furthermore, normalization of perfusion abnormalities in patients with depression after treatment was observed while no change in cerebral blood flow was noted in hypothyroidism. This implies that behavioral symptoms in depression may be mediated by different neural circuits from that seen in hypothyroidism [92].

## 13. Conclusion

Clinical investigators have long recognized the link between thyroid and depression. While patients with hypothyroidism commonly manifest features of depression, hyperthyroidism presents with a wider spectrum of neuropsychiatric symptoms including both depression and anxiety. On the other hand, most of the patients with primary depression have normal thyroid function. The mechanisms underlying the interaction between thyroid function and depression remain to be clarified and a causal relationship between the two cannot be established yet. A possible role for thyroid autoimmunity in the pathogenesis of depression can be elucidated. Screening patients presenting with depression for thyroid dysfunction seems reasonable particularly those with refractory symptoms. However, the use of thyroid hormones as an adjunct therapy to antidepressants in the absence of subclinical or clinical hypothyroidism should be further investigated. In addition, specifying a particular patient population that might benefit from this combination as determined by individual genetic variants should be addressed. The continuing research in the biochemical, genetic, and neuroimaging fields seems most promising in providing a deeper understanding of the thyroid-depression interactions.
